# Hypoxia Induces Dilated Cardiomyopathy in the Chick Embryo: Mechanism, Intervention, and Long-Term Consequences

**DOI:** 10.1371/journal.pone.0005155

**Published:** 2009-04-09

**Authors:** Andrei Tintu, Ellen Rouwet, Stefan Verlohren, Joep Brinkmann, Shakil Ahmad, Fatima Crispi, Marc van Bilsen, Peter Carmeliet, Anne Cathrine Staff, Marc Tjwa, Irene Cetin, Eduard Gratacos, Edgar Hernandez-Andrade, Leo Hofstra, Michael Jacobs, Wouter H. Lamers, Ingo Morano, Erdal Safak, Asif Ahmed, Ferdinand le Noble

**Affiliations:** 1 Laboratory for Angiogenesis and Cardiovascular Pathology, Max-Delbrueck-Center for Molecular Medicine, Berlin, Germany; 2 Department of Surgery, Cardiovascular Research Institute Maastricht, Maastricht University, Maastricht, the Netherlands; 3 Department of Vascular Surgery, Erasmus University Medical Center, Rotterdam, the Netherlands; 4 Department of Obstetrics, Charité University Medicine, Berlin, Germany; 5 Department of Reproductive & Vascular Biology, Centre for Cardiovascular Sciences, Institute for Biomedical Research, University of Birmingham, Birmingham, United Kingdom; 6 Department of Maternal-Fetal Medicine, Hospital Clinic, University of Barcelona, Barcelona, Spain; 7 Department of Physiology, Cardiovascular Research Institute Maastricht, Maastricht University, Maastricht, the Netherlands; 8 Center of Transgene Technology and Gene Therapy, University of Leuven, Leuven, Belgium; 9 Department of Obstetrics and Gynecology, Ulleval University Hospital, Oslo, Norway; 10 Institute of Cardiovascular Regeneration, Centre for Molecular Medicine, University of Frankfurt, Frankfurt, Germany; 11 Institute of Obstetrics and Gynecology, IRCCS Foundation Policlinico, Mangiagalli & Regina Elena, University of Milan, Milan, Italy; 12 Department of Cardiology, Cardiovascular Research Institute Maastricht, Maastricht University, Maastricht, the Netherlands; 13 Department of Embryology, Nutrition and Toxicology Research Institute Maastricht, Maastricht University, Maastricht, the Netherlands; 14 Research Group Molecular Muscle Physiology, Max-Delbrueck-Center for Molecular Medicine, Berlin, Germany; 15 Department of Cardiology, Franz Volhard Clinic, Helios Clinic Berlin-Buch, Charité University, Berlin, Germany; University of Cincinnati, United States of America

## Abstract

**Background:**

Intrauterine growth restriction is associated with an increased future risk for developing cardiovascular diseases. Hypoxia *in utero* is a common clinical cause of fetal growth restriction. We have previously shown that chronic hypoxia alters cardiovascular development in chick embryos. The aim of this study was to further characterize cardiac disease in hypoxic chick embryos.

**Methods:**

Chick embryos were exposed to hypoxia and cardiac structure was examined by histological methods one day prior to hatching (E20) and at adulthood. Cardiac function was assessed *in vivo* by echocardiography and *ex vivo* by contractility measurements in isolated heart muscle bundles and isolated cardiomyocytes. Chick embryos were exposed to vascular endothelial growth factor (VEGF) and its scavenger soluble VEGF receptor-1 (sFlt-1) to investigate the potential role of this hypoxia-regulated cytokine.

**Principal Findings:**

Growth restricted hypoxic chick embryos showed cardiomyopathy as evidenced by left ventricular (LV) dilatation, reduced ventricular wall mass and increased apoptosis. Hypoxic hearts displayed pump dysfunction with decreased LV ejection fractions, accompanied by signs of diastolic dysfunction. Cardiomyopathy caused by hypoxia persisted into adulthood. Hypoxic embryonic hearts showed increases in VEGF expression. Systemic administration of rhVEGF_165_ to normoxic chick embryos resulted in LV dilatation and a dose-dependent loss of LV wall mass. Lowering VEGF levels in hypoxic embryonic chick hearts by systemic administration of sFlt-1 yielded an almost complete normalization of the phenotype.

**Conclusions/Significance:**

Our data show that hypoxia causes a decreased cardiac performance and cardiomyopathy in chick embryos, involving a significant VEGF-mediated component. This cardiomyopathy persists into adulthood.

## Introduction

Epidemiological studies have shown that intrauterine stress, reflected by intrauterine growth restriction and low birth weight, increases the risk of developing cardiovascular diseases later in life [1]–[5]. The mechanisms underlying the relation between growth restriction and cardiovascular disease are unknown. A common cause of fetal growth restriction in clinical practice is intrauterine hypoxia due to uteroplacental insufficiency [6], [7].

In experimental models of embryonic hypoxia, it has been found that chronic hypoxia results in growth restriction, aortic remodeling, sympathetic hyperinnervation, and left ventricular (LV) pump dysfunction in the embryo [8]–[10]. The aim of the present study was to further characterize the effects of embryonic hypoxia on cardiac structure and function. In addition, we investigated the potential role of vascular endothelial growth factor (VEGF) herein. VEGF is a hypoxia-regulated cytokine that plays a pivotal role in angiogenesis, neurogenesis, and cardiac morphogenesis [11]–[13]. Studies in mice have indicated that even small perturbations in VEGF levels affect cardiovascular development and embryo survival [14]–[16].

We report that chronic hypoxia in chick embryos resulted in LV dilatation and several other structural and functional hallmarks of cardiomyopathy. Left ventricular dilatation and signs of cardiomyopathy persisted into adulthood and were, at least partly, secondary to increased VEGF levels.

## Results

### Hypoxia induces LV dilatation, cardiomyocyte apoptosis and signs of cardiomyopathy in E20 embryonic chick hearts

**Figure 1:**
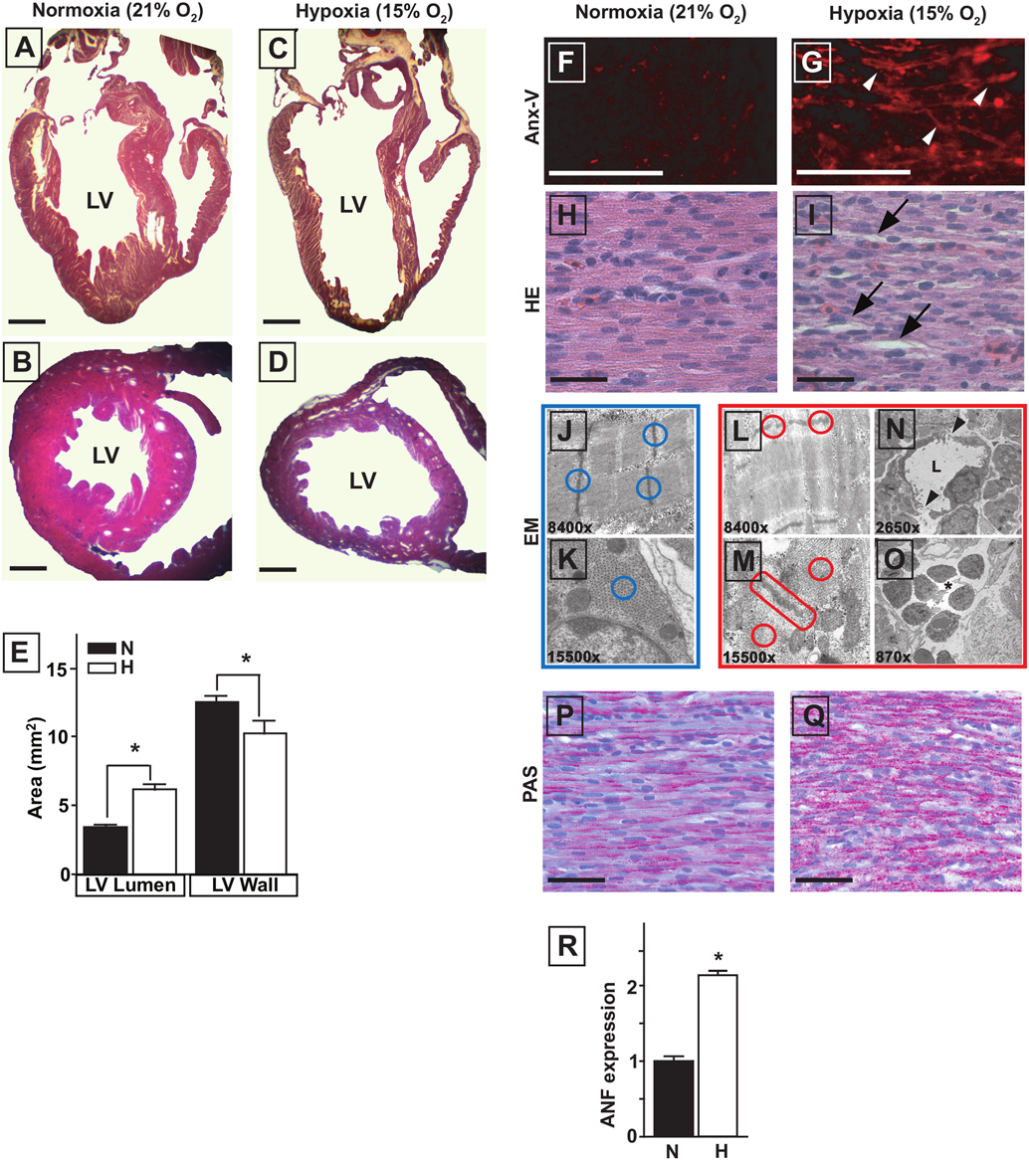
Histology of E20 chick embryo hearts. A to D, LV dilatation in hypoxic hearts. Scale bars represent 1 mm. E, cardiac morphometry showing a larger LV lumen and a thinner LV wall in hypoxia. F and G, increased number of apoptotic cardiomyocytes (arrowheads) in hypoxic hearts. Scale bars represent 100 µm. H and I, HE staining showing increased intracellular spaces (arrows) in the hypoxic hearts. J to O, ultrastructural analysis showing myofibrillar disarray, disruption of sarcomeric architecture (L and M, red circles), endothelial gaps in capillaries (N, arrowheads; L = lumen) and infiltration of inflammatory cells (O, asterisk) in hypoxic hearts. P and Q, PAS staining demonstrating increased glycogen deposition (red) in hypoxic hearts. R, Q-PCR showing increased ANF expression in hypoxic hearts. Data are shown as mean±SE; * P<0.05 Hypoxia versus Normoxia.

**Figure 2:**
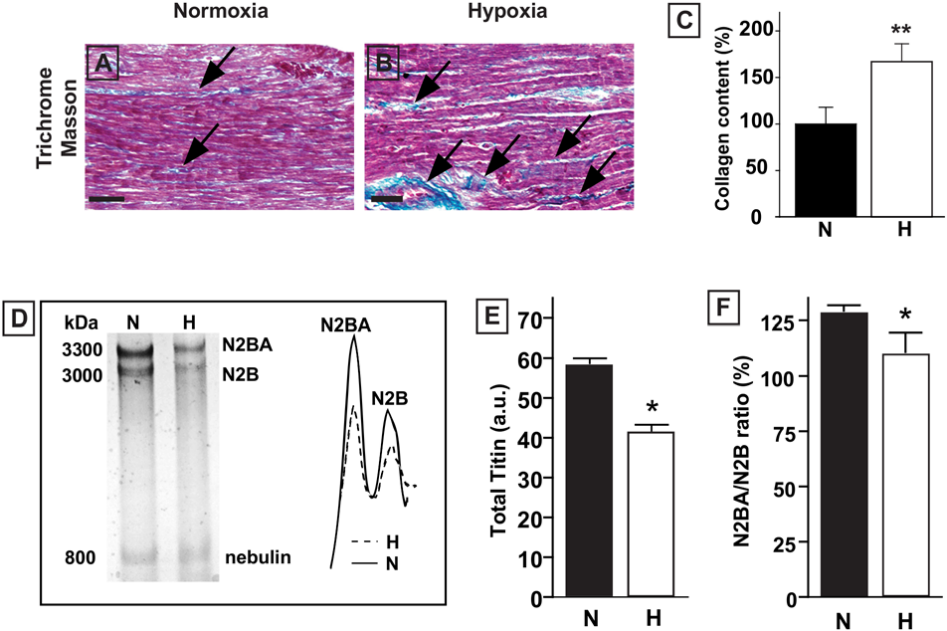
Cardiac content evaluation of E20 chick embryo hearts. A to C, trichrome Masson staining of left ventricular myocardium of E20 chick embryos showing increased interstitial collagen deposition in hypoxic hearts, both n = 6. Arrows denote collagen (blue). Scale bars represent 100 µm. Collagen content is significantly higher in hypoxic hearts (C). D, gel electrophoresis and densitometric analysis showing lowered titin protein levels and its isoforms in hypoxic E20 chick embryo hearts. E, densitometric quantification of total titin composition (N2BA+N2B) showing decreased levels of total titin in the hearts of the hypoxic chick embryos, n = 7 in both groups. F, the N2BA-to-N2B expression ratio is decreased in hypoxic embryonic hearts. N2BA: the large more compliant isoform (3300 kDa) of titin, N2B: the smaller stiffer isoform (3000 kDa) of titin, nebulin (800 kDa). Data are shown as mean±SE; * P<0.05, ** P<0.01 Hypoxia versus Normoxia; a.u. arbitrary units. (note: panel D does not show a molecular weight marker, because no suitable markers for the large molecular weight of titin are available).

Chick embryos that had been exposed to 15% O_2_ throughout embryonic development displayed lower arterial blood PO_2_ levels, elevated hematocrit, and lower total body, liver and heart weights as described previously [9], [10]. Hearts of hypoxic embryos displayed LV dilatation and loss of ventricular wall mass, without congenital cardiac defects (Figure 1A–E). Hearts of hypoxic chick embryos contained an increased number of apoptotic cardiomyocytes (Figure 1F–G) and histological analysis revealed disturbed arrangement of cardiomyocytes (Figure 1H–I). Cardiomyocyte degeneration, myofibrillar disarray with disruption of sarcomeric architecture, leaky blood vessels, and infiltration of inflammatory cells were observed at the ultrastructural level (Figure 1J–O). Hearts of hypoxic chick embryos also contained increased levels of glycogen (Figure 1P–Q), and expression levels of atrial natriuretic factor (ANF) were increased by a factor 2 (Figure 1R). Collagen content showed a 40% increase (Figure 2A–C), reflecting enhanced fibrosis. Levels of the sarcomeric proteins titin (Figure 2D–E) and myosin heavy chain (MHC, Table 1) were lowered in hypoxic embryonic chick hearts. The titin-to-MHC ratio was similar in normoxic and hypoxic hearts. Although the hypoxia-induced decrease in total titin content involved both the N2BA and the N2B isoform, the ratio between the isoforms changed significantly (Figure 2F, Table 1).

**Table 1:** Expression of total titin, titin isoforms, titin isoforms/total titin ratios and titin/myosin ratios.

	Normoxia (n = 7)	Hypoxia (n = 7)
**Total Titin (a.u.)**	58.4±3.2	41.4±4.2 *
**N2BA (a.u.)**	32.8±1.8	21.9±2.9 **
**N2B (a.u.)**	25.5±1.4	19.5±1.5 *
**Myosin (a.u.)**	115.3±2.4	100±1.8 **
**N2BA/Total Titin (%)**	56.2±0.5	51.9±1.9 *
**N2B/Total Titin (%)**	43.8±0.5	48.1±1.9 *
**N2BA/N2B (%)**	128.6±2.5	110.2±8.8 *
**N2B/N2BA (%)**	77.9±1.4	94.0±6.9 *
**Total Titin/Myosin (%)**	50.6±2.5	40.9±3.6

a.u.: arbitrary units.

* P<0.05.

** P<0.01.

### Hypoxia reduces cardiac performance in E20 chick embryos

**Figure 3:**
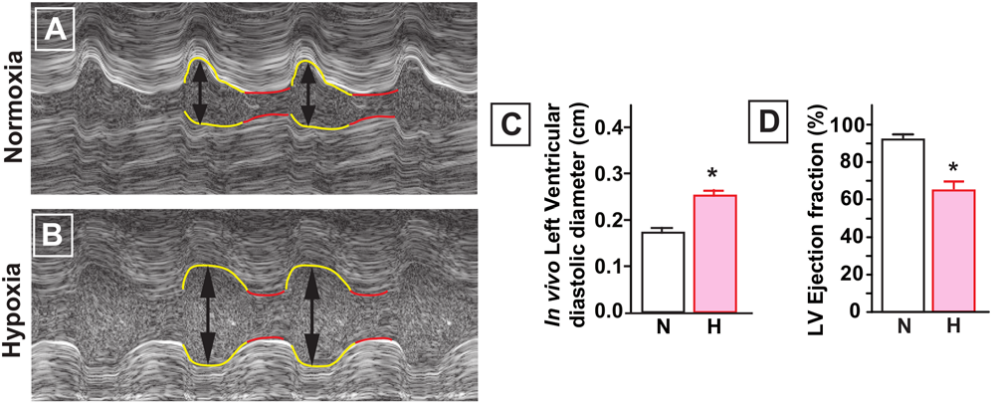
Echocardiography in E20 chick embryos. A and B, M-mode echo of normoxic and hypoxic embryos showing diastolic LV dilatation (arrows). C, diastolic left ventricular diameter is larger in the hypoxic embryos. D, hypoxic embryos demonstrate lower ejection fraction. Data are shown as mean±SE; * P<0.05 Hypoxia versus Normoxia.

**Figure 4:**
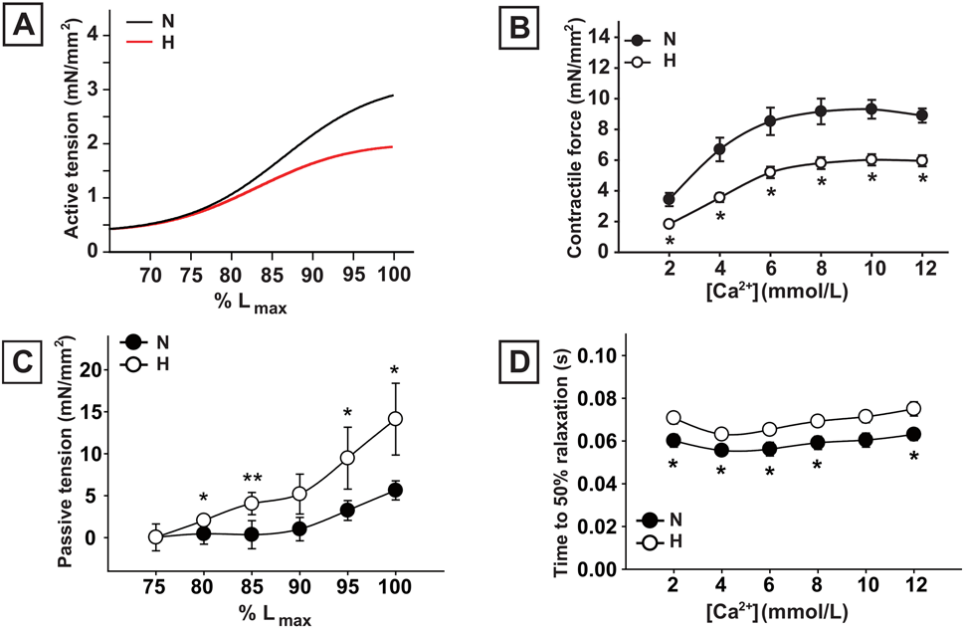
Contractile properties of LV muscle bundles in E20 chick embryos. A, the length-active tension relationship of LV muscle bundles is depressed in hypoxic E20 chick embryo hearts. B, LV contractility was lower in hypoxic LV muscle bundles. C, passive tension during stretching of LV muscle bundles is higher in hypoxic hearts, n = 6 and n = 8, respectively. D, the time to 50% relaxation is significantly prolonged in hypoxic as compared with normoxic hearts, both in response to increasing calcium concentrations, both n = 9. Data are shown as mean±SE; * P<0.05, ** P<0.01 Hypoxia versus Normoxia.

Assessing cardiac function *in vivo* by echocardiography revealed reduced LV ejection fractions and confirmed the presence of LV dilatation in hypoxic embryos (Figure 3A–D). Cardiac contractility was analyzed in isolated LV muscle bundles. The capacity to increase contractile force in response to stretch was significantly lower at 100% L_max_ in LV muscle bundles isolated from hypoxic hearts (Figure 4A). This indicates that the Frank-Starling mechanism is impaired. The calcium-mediated increase in contractile force development was approximately 40% lower in LV muscle bundles from the hypoxic embryos (Figure 4B). Passive tension, as determined by diastolic force during stretching, was significantly higher in hypoxic LV muscle bundles (Figure 4C). The time to 50% relaxation at varying calcium concentrations was significantly prolonged in the hypoxic group (Figure 4D).

### Cardiomyopathy caused by embryonic hypoxia persists in adult animals

**Figure 5:**
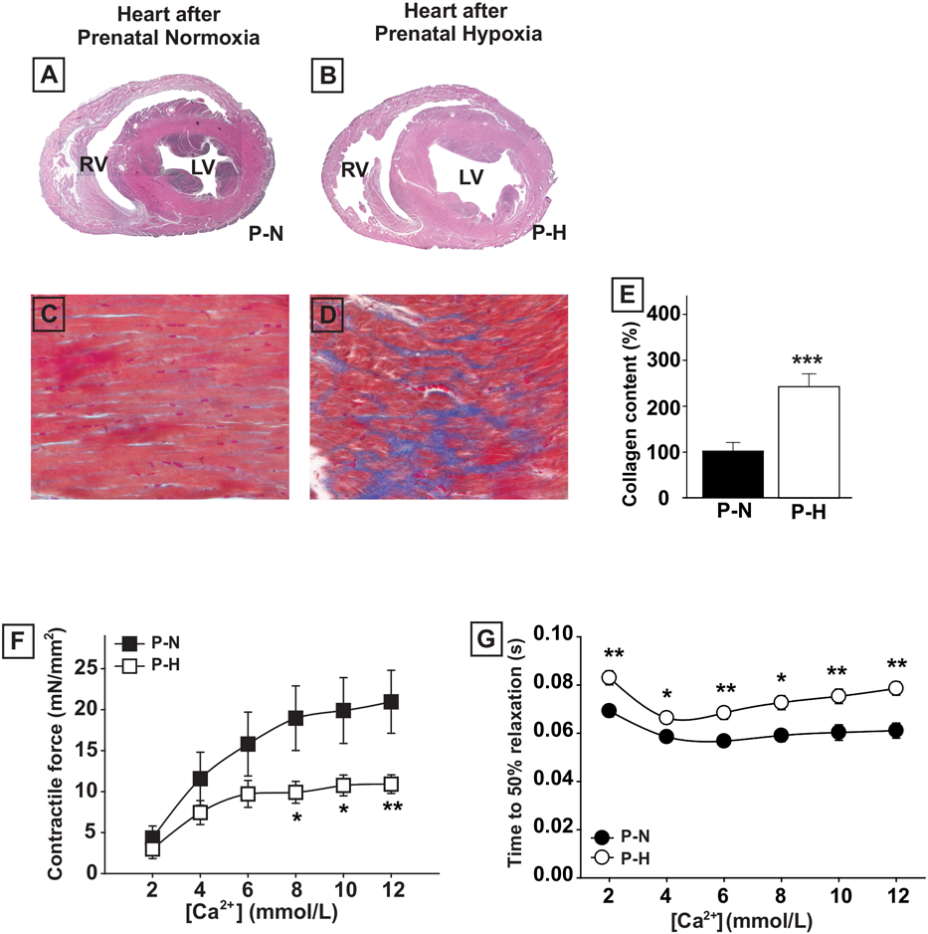
Hypoxia-induced dilated cardiomyopathy persists in adult chickens. A and B, morphology of adult hearts showing LV dilatation in adult chickens prenatally exposed to hypoxia (P-H) compared with adult chickens prenatally exposed to normoxia (P-N). C to E, trichrome Masson staining of left ventricular myocardium of adult chickens showing increased interstitial collagen deposition in hearts from adult animals prenatally exposed to hypoxia, both n = 6. Collagen content is significantly higher in hypoxic hearts (E). F, generated LV contractility was lower in muscle bundles from P-H animals. G, relaxation properties of LV muscle bundles isolated from adult chicken hearts. The time to 50% relaxation in response to increasing calcium concentrations is significantly prolonged in P-H animals, n = 7 in each group. Data are shown as mean±SE; * P<0.05, ** P<0.01 P-H versus P-N.

Adult chickens that had been exposed to hypoxia during embryonic development displayed severe LV dilatation in later life (Figure 5A, B). LV cavity cross-sectional area was approximately 4-fold larger than that of adult hearts prenatally exposed to normoxia (18.6±1.3 mm^2^ versus 4.6±0.6 mm^2^; *n* = 7; P<0.05). LV dilatation was accompanied by increases in myocardial collagen content (Figure 5C–E). Both the contractile responses and relaxation times to increasing calcium concentrations were significantly impaired in isolated LV muscle bundles from chickens prenatally exposed to hypoxia (Figure 5F–G).

### VEGF affects the contractility of isolated LV muscle bundles and cardiomyocytes

**Figure 6:**
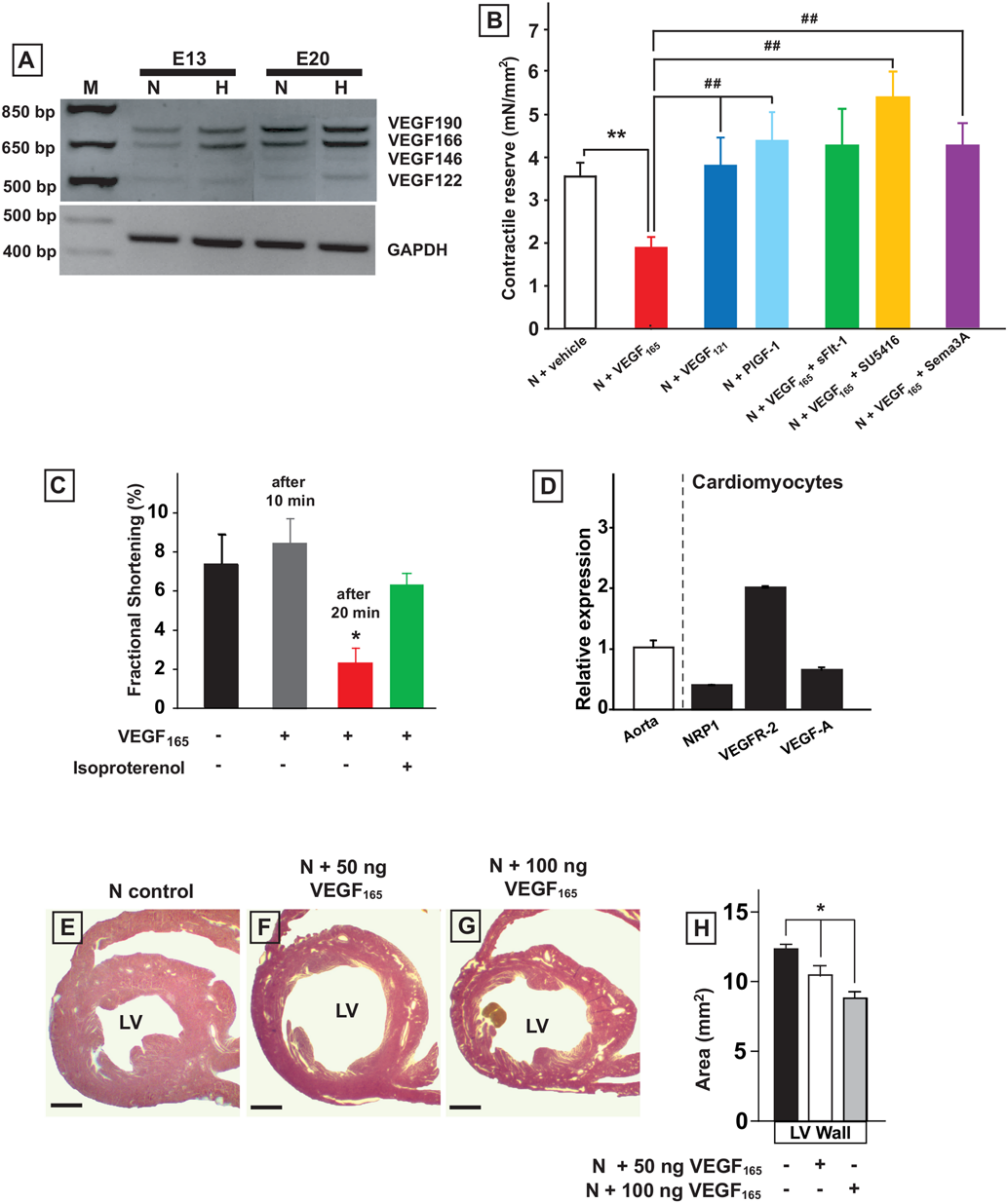
Influence of VEGF_165_ on the function and structure of the embryonic heart. A, PCR showing increased expression of VEGF_166_ and VEGF_190_ isoforms in the hypoxic heart at E13 and E20. B, contractile reserve of LV muscle bundles from normoxic E20 hearts after *ex vivo* incubation with VEGF_165_ (n = 6), VEGF_121_ (n = 7), PlGF-1 (n = 6), VEGF_165_+sFlt-1 (n = 6), VEGF_165_+SU5416 (n = 7), and VEGF_165_+Sema3A (n = 7). Only VEGF_165_ exerts a negative inotropic effect compared to vehicle (n = 13), which is abolished when co-incubated with sFlt-1, the VEGFR-2 inhibitor SU5416, or the NRP1 ligand Sema3A. C, VEGF_165_ impairs fractional shortening of isolated cardiomyocytes (n = 15). D, Q-PCR showing expression of VEGF-A, VEGFR-2, and NRP1 in isolated cardiomyocytes. E to H, *in vivo* treatment of normoxic embryos with VEGF_165_ leads to dose dependent LV dilatation. Data are shown as mean±SE; * P<0.05 VEGF_165_ versus vehicle *in vivo* incubated E20 normoxic chick embryos. ** P<0.01 VEGF_165_ versus vehicle *ex vivo* incubated E20 normoxic muscle bundles. ## P<0.01 versus VEGF_165_ *ex vivo* incubated E20 normoxic LV muscle bundles.

Hypoxic chick embryos showed an increased cardiac expression of the large VEGF-A isoforms VEGF_166_ and VEGF_190_ at both E13 and E20 (Figure 6A). To explore the potential role of VEGF as a mediator of cardiac dysfunction, we first tested the effects of different VEGF-A isoforms on LV muscle bundles and isolated cardiomyocytes. In LV muscle bundles from normoxic chick embryos, incubation with the rhVEGF_165_ isoform induced a severe reduction of the contractile reserve (Figure 6B). Neither rhVEGF_121_, the human equivalent of the smaller VEGF_122_ isoform that did not show increased expression in our model (Figure 6A), nor placental growth factor (rhPlGF-1), a second growth factor belonging to the VEGF protein family, exerted this effect (Figure 6B). Analogously, administration of rhVEGF_165_ to isolated cardiomyocytes caused a decrease in fractional shortening (Figure 6C), whereas rhPLGF-1 did not exert any effects (data not shown). The VEGF receptors VEGFR-2 and neuropilin-1 (NRP1) were demonstrated in isolated cardiomyocytes (Figure 6D). The negative inotropic effect of VEGF_165_ was abolished when muscle bundles were co-incubated with rhVEGF_165_ and sFlt-1 or with the VEGFR-2 tyrosine kinase inhibitor SU5416 [17] (Figure 6B). The used VEGF isoforms exhibit differential affinity to VEGFR-1, VEGFR-2 and the VEGF_165_ co-receptor NRP1. Since only VEGF_165_, but not VEGF_121_ or PlGF-1, binds to NRP1, we next investigated the potential role of NRP1 using the NRP1 ligand Semaphorin-3A (Sema3A). To achieve competitive antagonism of VEGF_165_ binding to NRP1, muscle bundles were co-incubated with Sema3A [18], which prevented the negative inotropic effect of VEGF_165_ (Figure 6B).

### VEGF is a mediator of cardiomypathy in the hypoxic chick embryo

**Figure 7:**
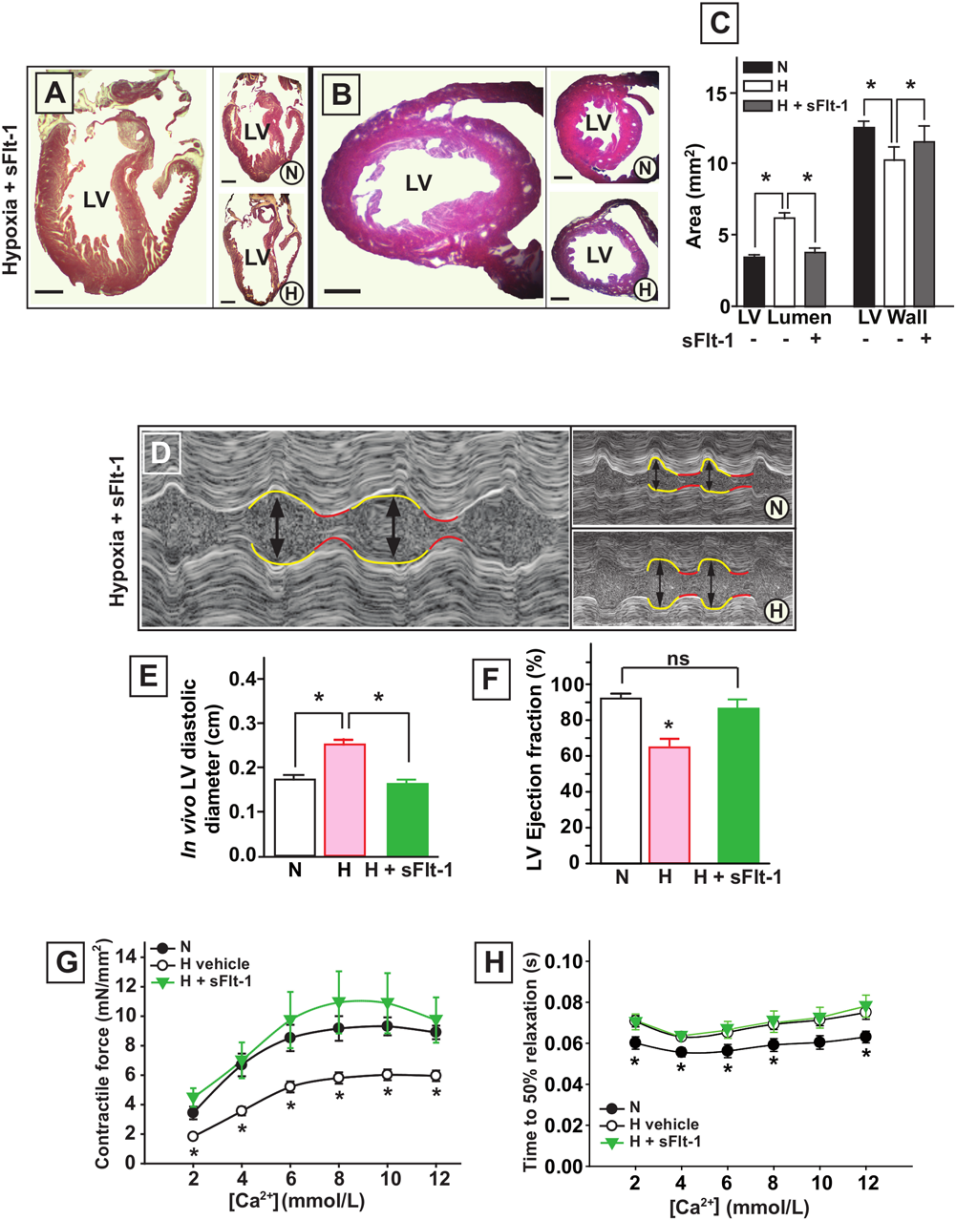
Treatment of hypoxic chick embryos with sFlt-1. A to C, LV dilatation is absent in hypoxic chick embryos treated with sFlt-1 as compared with normoxic (N) and hypoxic (H) controls. Scale bars represent 1 mm. D and E, M-mode echocardiography of normoxic and hypoxic embryos showing that LV dilatation (arrows, yellow lines) is prevented by sFlt-1 treatment. F, treatment of hypoxic embryos with sFlt-1 restores the LV ejection fraction. G, treatment of hypoxic embryos with sFlt-1 restores LV contractility as assessed in isolated muscle bundles. H, the time to 50% relaxation at increasing calcium concentrations of isolated LV muscle bundles shows no difference between sFlt-1 treated and vehicle treated hypoxic embryos, both n = 5. Data are shown as mean±SE; * P<0.05 sFlt-1 treated versus vehicle treated hypoxic embryos.

We next examined whether VEGF could induce cardiomyopathy *in vivo*. rhVEGF_165_ was systemically administered to normoxic chick embryos between E10 and E19. This resulted in LV dilatation and a dose-dependent loss of ventricular wall mass (Figure 6E–H). We tested whether lowering functional VEGF levels in hypoxic embryos influenced the development of cardiomyopathy in our model. The VEGF scavenger sFlt-1 was systemically administered to hypoxic embryos from E10 to E19. The increase in LV lumen diameter and loss of LV wall mass as observed in hypoxic embryos at E20 was thereby almost completely prevented (Figure 7A–E). Hearts from hypoxic embryos exposed to sFlt-1 were macroscopically indistinguishable from normoxic embryos and their LV ejection fraction, as assessed by echocardiography, was normalized (Figure 7F). Administration of sFlt-1 also restored contractility of isolated LV muscle bundles in E20 hypoxic embryos, to levels equaling the contractile responses to calcium of normoxic muscle bundles (Figure 7G). The time to 50% relaxation, however, was not influenced by sFlt-1 exposure, and remained at the level of hypoxic muscle bundles (Figure 7H).

## Discussion

Fetal hypoxia is a common clinical problem, frequently caused by uteroplacental insufficiency and disturbed blood supply to the fetus. Several studies have shown that hypoxia has significant impact on the developing heart in early developmental stages [8]–[10], [19]–[21]. Here, we demonstrated that embryonic hypoxia results in cardiac disease at both late embryonic stages and adulthood in a chick model. Furthermore, we identified a putative role for elevated cardiac VEGF_165_ levels in the etiology of hypoxia-initiated cardiac disease.

Hypoxic chick embryos displayed severe LV dilatation just prior to hatching, which was accompanied by a reduced LV wall mass, increased apoptosis, as well as a lowered cardiac content of the sarcomeric proteins MHC and titin. At the molecular level, we found increases in glycogen accumulation, gene induction of ANF and increased collagen contents, which are all linked to the development of cardiac hypertrophy and failure [22]. Our data suggest that the hypoxia-induced LV dilatation is associated with a loss of cardiomyocytes. This loss was estimated to amount approximately 9%, as evaluated by quantifying cells on tissue sections (data not shown).

Cardiac function upon chronic hypoxia was severely compromised in our chick embryo model. Systolic dysfunction was evidenced by a reduced LV ejection fraction in echocardiography and by significant decreases in contractile force development in LV muscle bundles isolated from hypoxic hearts. Moreover, we previously demonstrated cardiac dysfunction in an isolated heart perfusion system [9]. Additionally, muscle bundles from hypoxic hearts displayed an increase in passive tension upon stretching together with an increase in the time to 50% relaxation, indicating that impaired contractility was accompanied by increases in myocardial stiffness. Impaired left ventricular relaxation and reduced compliance are well known characteristics of cardiac diastolic dysfunction [23], and may be caused by abnormalities in the extracellular matrix or in the contractile apparatus of cardiomyocytes [24], [25]. The increase in the interstitial protein collagen and decrease in the sarcomeric proteins MHC and titin in hypoxic hearts might account for the observed changes [26]–[29]. We also found reduced titin N2BA-to-N2B expression ratios in hypoxic chick hearts. The shift in expression ratio of N2BA (the larger, more compliant isoform) towards N2B (the smaller, stiffer isoform) in the dilated hypoxic hearts will likely increase myocardial stiffness and impair cardiac compliance [30], [31].

Interestingly, adult chickens exposed to chronic hypoxia during embryonic development showed severely dilated hearts and impairment in the contractility of isolated LV muscle bundles. This strongly suggests that the embryonic hypoxia-induced contractile dysfunction is permanent. Moreover, the adult hearts displayed increased cardiac collagen contents and prolonged muscle bundle relaxation times, suggesting that the biomechanical alterations induced by embryonic hypoxia also persist into adulthood.

The increased expression of large VEGF-A isoforms (VEGF_166_ and VEGF_190_) in hypoxic embryonic hearts provided us the rationale for studying the role of this hypoxia-responsive cytokine. We show *in vivo* that both conditions of increased and decreased VEGF-A levels have an essential role in the origin of the cardiac disease. First, exposing normoxic embryos to rhVEGF_165_ initiated LV dilatation and loss of wall mass. Vice versa, scavenging VEGF-A with sFlt-1 in hypoxic embryos prevented LV dilatation and loss of wall mass, and restored LV ejection fraction and muscle bundle contractility to levels resembling normoxic embryos. In contrast, diastolic properties remained unchanged in E20 hypoxic embryos after sFlt-1 treatment, suggesting separate mechanisms for systolic and diastolic dysfunction. It has been reported that reduced cardiac VEGF signaling interferes with myocardial angiogenesis, resulting in local ischemia, which triggers cardiomyocyte damage and heart failure [32]. In our experiments, we observed the opposite effect; reducing cardiac VEGF levels with sFlt-1 prevented cardiac disease in hypoxic embryos. The number of arterioles quantified in LV myocardium was similar in sFlt-1 and vehicle-treated hypoxic embryos (data not shown), suggesting that the effect of sFlt-1 was independent from the cardiac vasculature.

Both experiments in isolated LV muscle bundles and in cardiomyocytes demonstrated that specifically the VEGF_165_ isoform exerts a negative inotropic effect. We found that chick embryo cardiomyocytes express the VEGF receptors VEGFR-2 and NRP1. Exposure of LV muscle bundles to SU5416, a selective inhibitor of VEGFR-2 signaling [17] or to Sema3A, a competitive antagonist of VEGF_165_ binding to NRP1 [18] blocked the negative inotropic effect of rhVEGF_165_. Taken together, our experiments suggest that the large VEGF_165_ isoform, through binding to VEGFR-2 and NRP1, directly affects the contractile properties of cardiomyocytes, thereby initiating LV dilatation and cardiac disease.

A recent study on intrauterine growth restriction in human fetuses indicated that increases in cardiac afterload and end-diastolic filling pressure were the most important effects on the cardiovascular system. Also evidenced were disturbances in arterial and cardiac compliance. These alterations may potentially program the growth-retarded fetus for cardiac hypertrophy and hypertension [33]. Aortic remodeling, sympathetic hyperinnervation, and alterations of vascular tone in hypoxic embryos [8]–[10] may all contribute to increased cardiac afterload. In the present study we identified VEGF as a trigger for cardiac disease in a hypoxic chick embryo model. An afterload-independent effect of VEGF in the development of cardiac disease is supported by the finding that administration of VEGF to normoxic embryos, which theoretically decreases cardiac afterload, also induced LV dilatation and contractile dysfunction. Our *in vitro* experiments suggest that elevated cardiac VEGF_165_ levels directly affect cardiomyocyte function and warrant further research on the specific mechanism.

## Methods

### Ethics Statement

All experiments were approved by the Institutional Animal Care and Use Committee of the Maastricht University and complied with the principles of laboratory animal care.

### Animals

Fertile White Leghorn eggs were incubated at 21% O_2_ (normoxia; N) or 15% O_2_ (hypoxia; H) throughout embryonic development as described previously [9], [10]. Experiments were performed between E10-E20 of the 21-day incubation period. To evaluate the effects of embryonic hypoxia on the adult heart, normoxic and hypoxic chick embryos were allowed to hatch at E21 and both groups were raised under normal ambient oxygen levels of 21% O_2_. Structural and functional analysis of adult hearts was performed at 8 months after hatching. Chick embryos and adult chickens were sacrificed by decapitation.

### Cardiac histology

Hearts were fixed during diastole using 100 mmol/L CdCl_2_. LV cross-sectional area was measured on formalin-fixed sections (ImageJ, National Institutes of Health). Consecutive paraffin-embedded 5-µm sections were stained with hematoxylin and eosin (HE), PAS, and trichrome Masson to visualize tissue fibrosis. The area of fibrotic tissue was quantified by computer software (Quantimet 570, Leica). Ultrastructural analysis of epoxy resin embedded hearts was performed on 70-nm sections.

### Titin protein levels

Hearts from normoxic and hypoxic E20 embryos were excised, snap-frozen, and stored at −80°C. Left ventricular tissue samples were homogenized in 4 mL SET-buffer (in mol/L 0.25 sucrose, 0.01 Tris-HCl, 0.002 Na_2_-EDTA; pH 7.4). Leupeptin (0.5 mmol/L) was added to prevent protein degradation. Protein content was determined by the Micro BCA method (Pierce, Rockford, USA). Samples (20 µg protein) were loaded on a 2% SDS-polyacrylamide slab gel reinforced with agarose and electrophorized using a Mini-PROTEAN II electrophoresis system (BioRad, Hercules, USA). Fairbanks running buffer was used for electrophoresis and the conditions were 7.5 mA/gel for 30 min and 15 mA/gel for 5 hours [34]. Gels were stained with Coomassie Brilliant Blue R-250. The integrated optical density (OD) of N2BA titin, N2B titin, total titin (N2BA+N2B), and myosin heavy chain (MHC) were determined at each lane.

### Embryonic echocardiography

Echocardiography was performed in vivo at E20. The animals were carefully hatched with as less blood loss as possible and immediately investigated. Heart rates and temperatures were monitored (Model THM100, Indus Instruments, Houston, Texas), and heating was adjusted to maintain a body temperature between 36 and 38°C. All feathers were removed from the abdomen by gentle shaving. Pre-warmed gel was applied to the precordium as an ultrasound coupling medium (Parker Laboratory, Orange, NJ). Chick embryo hearts were imaged transcutaneously using the ultrasound biomicroscope (UBM) and a 30 MHz or 40 MHz transducer operating at 30 frames/s (Model Vevo 660, VisualSonics Inc., Toronto, Canada). Generally, the heart was first imaged in the two-dimensional (2-D) mode in the short-axis view. Images from this view were used to measure left ventricular (LV) cross-sectional area. Images were stored on harddisk for review and analysis. LV end-diastolic diameter (LVEDD) was measured from the M-mode tracings of the maximum chamber cavity. All measurements were performed from leading edge to leading edge according to the American Society of Echocardiography guidelines. Ejection fraction was calculated from the LV cross-sectional area (2-D short-axis view) using the equation EF (%) = [(LVDA−LVSA)/LVDA]×100; where LVDA is LV diastolic area and LVSA is LV systolic area. All primary measurements such as LV wall thickness, dimensions, and cross-sectional areas were traced manually and digitized by goal-directed, diagnostically driven software installed within the echocardiograph. Three beats were averaged for each measurement.

### Isolated LV muscle bundles

Hearts were in situ perfused with 0.2 mL heparin (5000 U/mL), and immediately after surgical resection placed in a Ca^2+^-free solution (in mmol/L: 135 NaCl, 5.4 KCl, 0.33 NaH_2_PO_4_, 10 HEPES, 10 C_6_H_12_O_6_xH_2_O, 1 MgCl_2_, and 30 2,3-Butanedione 2-monoxime, pH 7.4, gassed with 100% O_2_). A thin subendocardial LV muscle bundle was excised in parallel with fiber direction, mounted in an organ bath with HEPES buffer (in mmol/L: 10 HEPES, 2 Ca^2+^, pH 7.4, gassed with 100% O_2_, 37°C) between a rigid hook and a force transducer connected to a micromanipulator for length adjustment, as described [10], [35].

After an equilibration period of 10 minutes, the muscle bundle was paced by external field stimulation with rectangular pulses (5 ms, 5% to 10% above threshold) at a frequency of 1 Hz. This resulted in isometric contraction of the fiber. Subsequently, the muscle was stretched in steps of 218 µm. Passive tension (mN) was determined by measuring force during diastole, i.e. unstimulated force, at incremental bundle lengths [36]. The length-active tension relation, i.e. Frank-Starling relation, was assessed by measuring active force generation during field stimulation at incremental bundle lengths. Bundle length was expressed as a percentage of L_max_. Generated force was defined as peak systolic minus diastolic force and normalized to cross-sectional area (mN/mm^2^) by optical measurement of the smallest diameter of the bundle assuming cylindrical geometry. Muscle bundles were stretched until the fiber length was reached which provided maximal active force generation (100% L_max_). For standardization purposes, all further experiments were performed at 100% L_max_ after a resting period of 5 minutes. Isometric contractile force development was measured in response to increasing extracellular calcium (2–12 mmol/L). Relaxation properties of the LV muscle bundles were assessed by measuring the time to 50% relaxation during diastole in these experiments.

### VEGF and cardiac performance

In separate experiments, contractility of LV muscle bundles from normoxic E20 chick embryos was measured after 4 hours incubation with: 100 ng/mL rhVEGF_165_ [37], 600 ng/mL rhVEGF_121_ [37], 600 ng/mL placental growth factor-1 (rhPlGF-1), 100 ng/mL rhVEGF_165_+130 ng/mL sFlt-1, 100 ng/mL rhVEGF_165_+130 ng/mL rhSema3A (all R&D Systems), 100 ng/mL rhVEGF_165_+50 µmol/L SU5416 (Sigma), or HEPES buffer alone.

The effect of VEGF was further defined *in vitro* by measuring fractional shortening of isolated rat cardiomyocytes in response to rhVEGF_165_ or rhPlGF-1 (500 ng/mL) using a Ionoptix Contractility System (Ionoptix, Milton, MA, USA) [38].

### Molecular analysis

Real-time PCR for atrial natriuretic factor (ANF), VEGF, VEGFR-2, and NRP1 was conducted using the My iQ iCycler and iQ SYBR Green Supermix (both Bio-Rad) (Table 2). Semi-quantitative PCR analysis was performed to detect the expression of VEGF-A mRNA splice variant isoforms [39]. Cardiomyocyte apoptosis was assessed by *in vivo* molecular imaging using annexin-V [40].

**Table 2:** Primers used for Real-Time PCR.

Quantitative Real-Time PCR Primers			
Gene	Accession Number	Sense Primer	Antisense Primer
**ANF**	NM_204925	AGCAGAGCCAACCCCATCTA	TTGGACTCCAGGGCTTCAAT
**VEGF-A**	AB011078	TCTGCAGGACAATTGAGACCC	AACCCGCACATCTCATCAGAG
**VEGFR-2**	X83288	TGGGATGGTTCTTGCATCTGA	GACTCATTGCTTTTGCTGGGC
**NRP1**	D45416	AACCAGGCAATGTCCTGAAG	CAACTCCACAAATGGCTCCT
**18S**	AF173612	ATGGCCGTTCTTAGTTGGTG	GAACGCCACTTGTCCCTCTA

### In vivo application of VEGF_165_ or sFlt-1

To test the involvement of VEGF_165_ in hypoxia-induced LV dilatation, normoxic chick embryos received a daily systemic dose of rhVEGF_165_ (R&D Systems, 50 or 100 ng/day) between E10 and E19. Vice versa, hypoxic embryos were treated with sFlt-1 (both R&D Systems, 13 ng/day) between E10 and E19 [41]. Controls received saline. The solutions were administered by daily 0.1 mL bolus injections in the air space of the egg [42]. Cardiac morphology and function were analyzed at E20.

### Data analysis

Data are expressed as mean±SE. The term n refers to the number of animals. Multiple comparisons were made using the non-parametric Mann-Whitney U test. Statistical significance was defined as P<0.05.

## References

[1] Gillman MW (2005). Developmental origins of health and disease.. N Engl J Med.

[2] Ingelfinger JR (2004). Pathogenesis of perinatal programming.. Curr Opin Nephrol Hypertens.

[3] Louey S, Thornburg KL (2005). The prenatal environment and later cardiovascular disease.. Early Hum Dev.

[4] Barker DJ, Osmond C, Golding J, Kuh D, Wadsworth ME (1989). Growth in utero, blood pressure in childhood and adult life, and mortality from cardiovascular disease.. BMJ.

[5] Leeson CP, Kattenhorn M, Morley R, Lucas A, Deanfield JE (2001). Impact of low birth weight and cardiovascular risk factors on endothelial function in early adult life.. Circulation.

[6] Nicolaides KH, Economides DL, Soothill PW (1989). Blood gases, pH, and lactate in appropriate- and small-for-gestational-age fetuses.. Am J Obstet Gynecol.

[7] Pardi G, Cetin I, Marconi AM, Lanfranchi A, Bozzetti P (1993). Diagnostic value of blood sampling in fetuses with growth retardation.. N Engl J Med.

[8] Morrison JL, Botting KJ, Dyer JL, Williams SJ, Thornburg KL (2007). Restriction of placental function alters heart development in the sheep fetus.. Am J Physiol Regul Integr Comp Physiol.

[9] Rouwet EV, Tintu AN, Schellings MW, van Bilsen M, Lutgens E (2002). Hypoxia induces aortic hypertrophic growth, left ventricular dysfunction, and sympathetic hyperinnervation of peripheral arteries in the chick embryo.. Circulation.

[10] Tintu AN, Noble FA, Rouwet EV (2007). Hypoxia disturbs fetal hemodynamics and growth.. Endothelium.

[11] Dor Y, Camenisch TD, Itin A, Fishman GI, McDonald JA (2001). A novel role for VEGF in endocardial cushion formation and its potential contribution to congenital heart defects.. Development.

[12] Ferrara N, Gerber HP, LeCouter J (2003). The biology of VEGF and its receptors.. Nat Med.

[13] Giordano FJ, Gerber HP, Williams SP, VanBruggen N, Bunting S (2001). A cardiac myocyte vascular endothelial growth factor paracrine pathway is required to maintain cardiac function.. Proc Natl Acad Sci U S A.

[14] Carmeliet P, Ferreira V, Breier G, Pollefeyt S, Kieckens L (1996). Abnormal blood vessel development and lethality in embryos lacking a single VEGF allele.. Nature.

[15] Miquerol L, Langille BL, Nagy A (2000). Embryonic development is disrupted by modest increases in vascular endothelial growth factor gene expression.. Development.

[16] Feucht M, Christ B, Wilting J (1997). VEGF induces cardiovascular malformation and embryonic lethality.. Am J Pathol.

[17] Fong TA, Shawver LK, Sun L, Tang C, App H (1999). SU5416 is a potent and selective inhibitor of the vascular endothelial growth factor receptor (Flk-1/KDR) that inhibits tyrosine kinase catalysis, tumor vascularization, and growth of multiple tumor types.. Cancer Res.

[18] Miao HQ, Soker S, Feiner L, Alonso JL, Raper JA (1999). Neuropilin-1 mediates collapsin-1/semaphorin III inhibition of endothelial cell motility: functional competition of collapsin-1 and vascular endothelial growth factor-165.. J Cell Biol.

[19] Ream M, Ray AM, Chandra R, Chikaraishi DM (2008). Early fetal hypoxia leads to growth restriction and myocardial thinning.. Am J Physiol Regul Integr Comp Physiol.

[20] Sharma SK, Lucitti JL, Nordman C, Tinney JP, Tobita K (2006). Impact of hypoxia on early chick embryo growth and cardiovascular function.. Pediatr Res.

[21] Villamor E, Kessels CG, Ruijtenbeek K, van Suylen RJ, Belik J (2004). Chronic in ovo hypoxia decreases pulmonary arterial contractile reactivity and induces biventricular cardiac enlargement in the chicken embryo.. Am J Physiol Regul Integr Comp Physiol.

[22] Gupta S, Das B, Sen S (2007). Cardiac hypertrophy: mechanisms and therapeutic opportunities.. Antioxid Redox Signal.

[23] Katz AM, Zile MR (2006). New molecular mechanism in diastolic heart failure.. Circulation.

[24] Zile MR, Brutsaert DL (2002). New concepts in diastolic dysfunction and diastolic heart failure: Part II: causal mechanisms and treatment.. Circulation.

[25] Zile MR, Brutsaert DL (2002). New concepts in diastolic dysfunction and diastolic heart failure: Part I: diagnosis, prognosis, and measurements of diastolic function.. Circulation.

[26] Nagueh SF, Shah G, Wu Y, Torre-Amione G, King NM (2004). Altered titin expression, myocardial stiffness, and left ventricular function in patients with dilated cardiomyopathy.. Circulation.

[27] Schaper J, Froede R, Hein S, Buck A, Hashizume H (1991). Impairment of the myocardial ultrastructure and changes of the cytoskeleton in dilated cardiomyopathy.. Circulation.

[28] Hein S, Scholz D, Fujitani N, Rennollet H, Brand T (1994). Altered expression of titin and contractile proteins in failing human myocardium.. J Mol Cell Cardiol.

[29] Hein S, Kostin S, Heling A, Maeno Y, Schaper J (2000). The role of the cytoskeleton in heart failure.. Cardiovasc Res.

[30] Wu Y, Bell SP, Trombitas K, Witt CC, Labeit S (2002). Changes in titin isoform expression in pacing-induced cardiac failure give rise to increased passive muscle stiffness.. Circulation.

[31] van Heerebeek L, Borbely A, Niessen HW, Bronzwaer JG, van der Velden J (2006). Myocardial structure and function differ in systolic and diastolic heart failure.. Circulation.

[32] Shiojima I, Sato K, Izumiya Y, Schiekofer S, Ito M (2005). Disruption of coordinated cardiac hypertrophy and angiogenesis contributes to the transition to heart failure.. J Clin Invest.

[33] Verburg BO, Jaddoe VW, Wladimiroff JW, Hofman A, Witteman JC (2008). Fetal hemodynamic adaptive changes related to intrauterine growth: the Generation R Study.. Circulation.

[34] Tatsumi R, Hattori A (1995). Detection of giant myofibrillar proteins connectin and nebulin by electrophoresis in 2% polyacrylamide slab gels strengthened with agarose.. Anal Biochem.

[35] Nosek TM, Fogaca RT, Hatcher CJ, Brotto MA, Godt RE (1997). Effect of cardiac neural crest ablation on contractile force and calcium uptake and release in chick heart.. Am J Physiol.

[36] Holubarsch C, Ruf T, Goldstein DJ, Ashton RC, Nickl W (1996). Existence of the Frank-Starling mechanism in the failing human heart. Investigations on the organ, tissue, and sarcomere levels.. Circulation.

[37] Yue X, Tomanek RJ (2001). Effects of VEGF(165) and VEGF(121) on vasculogenesis and angiogenesis in cultured embryonic quail hearts.. Am J Physiol Heart Circ Physiol.

[38] Haase H, Dobbernack G, Tünnemann G, Karczewski P, Cardoso C (2006). Minigenes encoding N-terminal domains of human cardiac myosin light chain-1 improve heart function of transgenic rats.. FASEB Journal.

[39] Sugishita Y, Watanabe M, Fisher SA (2004). Role of myocardial hypoxia in the remodeling of the embryonic avian cardiac outflow tract.. Dev Biol.

[40] Dumont EA, Reutelingsperger CP, Smits JF, Daemen MJ, Doevendans PA (2001). Real-time imaging of apoptotic cell-membrane changes at the single-cell level in the beating murine heart.. Nat Med.

[41] Sugimoto H, Hamano Y, Charytan D, Cosgrove D, Kieran M (2003). Neutralization of circulating vascular endothelial growth factor (VEGF) by anti-VEGF antibodies and soluble VEGF receptor 1 (sFlt-1) induces proteinuria.. J Biol Chem.

[42] Stewart DE, Kirby ML (1985). Endogenous tyrosine hydroxylase activity in the developing chick heart: a possible source of extraneuronal catecholamines.. J Mol Cell Cardiol.

